# Novel ketone diet enhances physical and cognitive performance

**DOI:** 10.1096/fj.201600773R

**Published:** 2016-08-15

**Authors:** Andrew J. Murray, Nicholas S. Knight, Mark A. Cole, Lowri E. Cochlin, Emma Carter, Kirill Tchabanenko, Tica Pichulik, Melanie K. Gulston, Helen J. Atherton, Marie A. Schroeder, Robert M. J. Deacon, Yoshihiro Kashiwaya, M. Todd King, Robert Pawlosky, J. Nicholas P. Rawlins, Damian J. Tyler, Julian L. Griffin, Jeremy Robertson, Richard L. Veech, Kieran Clarke

**Affiliations:** * Department of Physiology, Anatomy, and Genetics, University of Oxford, Oxford, United Kingdom;; † Department of Physiology, Development, and Neuroscience, University of Cambridge, Cambridge, United Kingdom;; ‡ Department of Chemistry, University of Oxford, Oxford, United Kingdom;; § Department of Biochemistry, University of Cambridge, Cambridge, United Kingdom;; ¶ Cambridge Systems Biology Centre, University of Cambridge, Cambridge, United Kingdom;; ‖ Department of Experimental Psychology, University of Oxford, Oxford, United Kingdom;; # Laboratory of Metabolic Control, National Institutes of Health, National Institute on Alcohol Abuse and Alcoholism, Rockville, Maryland, USA

**Keywords:** metabolism, muscle, energetics, exercise, heart

## Abstract

Ketone bodies are the most energy-efficient fuel and yield more ATP per mole of substrate than pyruvate and increase the free energy released from ATP hydrolysis. Elevation of circulating ketones *via* high-fat, low-carbohydrate diets has been used for the treatment of drug-refractory epilepsy and for neurodegenerative diseases, such as Parkinson’s disease. Ketones may also be beneficial for muscle and brain in times of stress, such as endurance exercise. The challenge has been to raise circulating ketone levels by using a palatable diet without altering lipid levels. We found that blood ketone levels can be increased and cholesterol and triglycerides decreased by feeding rats a novel ketone ester diet: chow that is supplemented with (*R)*-3-hydroxybutyl (*R*)-3-hydroxybutyrate as 30% of calories. For 5 d, rats on the ketone diet ran 32% further on a treadmill than did control rats that ate an isocaloric diet that was supplemented with either corn starch or palm oil (*P* < 0.05). Ketone-fed rats completed an 8-arm radial maze test 38% faster than did those on the other diets, making more correct decisions before making a mistake (*P* < 0.05). Isolated, perfused hearts from rats that were fed the ketone diet had greater free energy available from ATP hydrolysis during increased work than did hearts from rats on the other diets as shown by using [^31^P]-NMR spectroscopy. The novel ketone diet, therefore, improved physical performance and cognitive function in rats, and its energy-sparing properties suggest that it may help to treat a range of human conditions with metabolic abnormalities.—Murray, A. J., Knight, N. S., Cole, M. A., Cochlin, L. E., Carter, E., Tchabanenko, K., Pichulik, T., Gulston, M. K., Atherton, H. J., Schroeder, M. A., Deacon, R. M. J., Kashiwaya, Y., King, M. T., Pawlosky, R., Rawlins, J. N. P., Tyler, D. J., Griffin, J. L., Robertson, J., Veech, R. L., Clarke, K. Novel ketone diet enhances physical and cognitive performance.

Food nutrients release metabolic energy as they are broken down into 2-carbon fragments and combusted to CO_2_ in the citric acid cycle (1). The reducing equivalents that are produced undergo a series of redox reactions in the electron transport system, ultimately converting O_2_ to H_2_O, thereby creating a proton gradient between mitochondria and cytosol (2), the energy of which is preserved in the pyrophosphate bond of ATP. However, different food nutrients, when catabolized to C_2_ units, release differing amounts of energy depending on their degree of oxidation and thus produce differing amounts of ATP per mole of substrate or ATP with a different free energy of hydrolysis, Δ*G,* by altering the relative concentrations of ATP, ADP, and phosphocreatine (PCr) in tissue.

Elevation of ketone body levels increases the energy of ATP hydrolysis by reducing the mitochondrial NAD couple and oxidizing the coenzyme Q couple, which thereby increases the redox span between complex I and complex II of the mitochondrial electron transport chain (3). Combustion of the end-glycolytic substrate, pyruvate, produces 10 ATP, whereas combustion of the ketone body, d-β-hydroxybutyrate, produces 13 ATP (4), which thereby increases the efficiency of hydraulic work in the working, perfused rat heart by ∼30% compared with oxidation of pyruvate (5). Ketones, as the most energy-efficient fuel, may therefore be beneficial for muscle and brain in times of stress, such as during endurance exercise (6).

Mild ketosis that results from the conversion of free fatty acids to ketone bodies in the liver is a physiologic response to prolonged fasting in humans (7), in whom elevated blood ketone bodies replace glucose as the major energy substrate for the brain (8). Therapeutically, elevation of circulating ketone concentrations by feeding high-fat, low-carbohydrate diets has been used for more than a century for treatment of drug-refractory epilepsy (9) and, more recently, for neurodegenerative diseases, such as Parkinson’s disease (10). The usefulness of these high-fat diets, however, is limited by poor patient tolerance and elevation of blood lipids, which offset many of the benefits of ketone metabolism (11).

Feeding a high-fat, low-carbohydrate ketogenic diet (9) increases plasma cholesterol and free fatty acids (11, 12). Both are undesirable: the former from an atherogenic point of view and the latter because elevated free fatty acids activate peroxisome proliferator-activated receptor transcription factors, which thereby increases uncoupling proteins (UCPs) and induces metabolic inefficiency (13, 14). Furthermore, short-term consumption of a high-fat diet impaired exercise tolerance and spatial working memory in rats (15), whereas in humans, consumption of a high-fat, low-carbohydrate ketogenic diet impaired whole-body exercise efficiency (16), cardiac high-energy phosphate metabolism (17), and cognitive function (16, 17).

The challenge, therefore, has been to elevate circulating ketone levels by using a palatable diet without altering lipid levels and thereby negating many of the desirable effects of ketone metabolism (6). Consequently, we made an orally absorbable form of ketone body by transesterifying ethyl (*R*)-3-hydroxybutyrate with (*R)*-1,3-butanediol by using a solid-supported lipase. The rationale behind our development of a ketone ester was the desire to feed an orally absorbable form of ketone without administering the cation load or acidosis that would accompany the feeding of a ketone salt (*e.g.,* Na-hydroxybutyrate) or free acid (β-hydroxybutyric acid). *(R)*-1,3-Butanediol was chosen because it is converted to acetoacetate and d-β-hydroxybutyrate in the liver (18); therefore, (*R)*-3-hydroxybutyl (*R*)-3-hydroxybutyrate would yield only ketone bodies upon hydrolysis and oxidation (**Fig. 1*A***). (*R*)-3-Hydroxybutyl (*R*)-3-hydroxybutyrate is palatable and nontoxic in rats (19) and humans (20).

**Figure 1.**
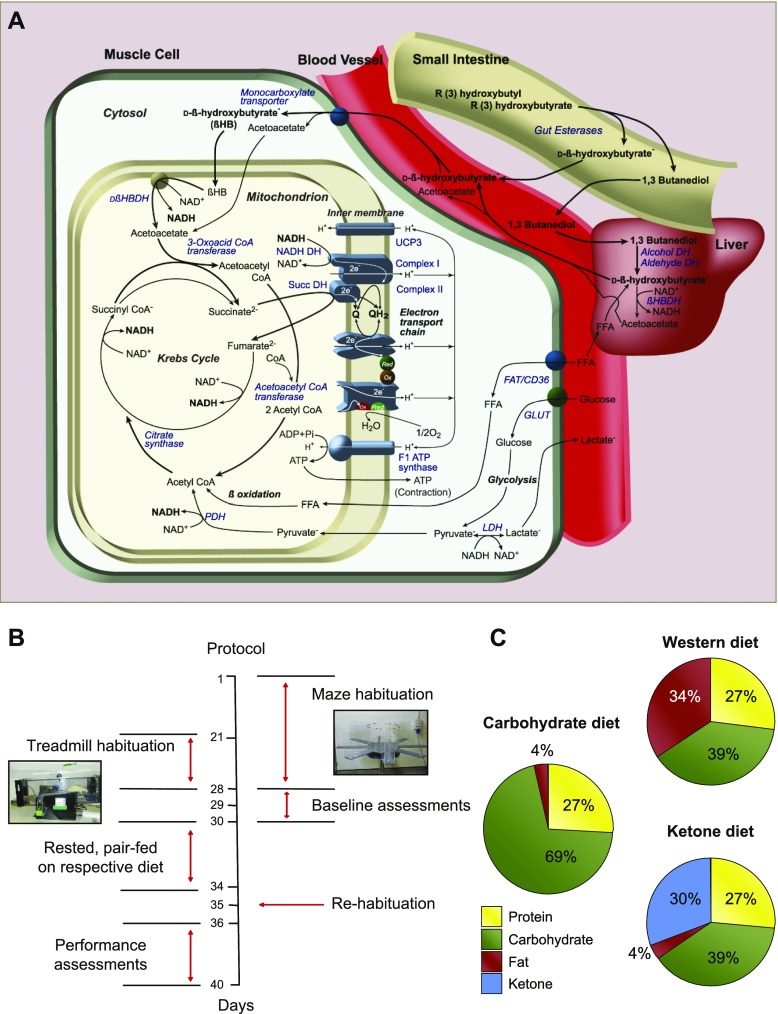
(*R*)-3-Hydroxybutyl (*R*)-3-hydroxybutyrate metabolism, experimental protocol and diets. *A*) (*R*)-3-Hydroxybutyl (*R*)-3-hydroxybutyrate, synthesized *via* a transesterification reaction, is hydrolyzed by gut esterases to yield absorbable d-β-hydroxybutyrate and (*R*)-1,3-butanediol. In the liver, (*R*)-1,3-butanediol is converted to acetoacetate and d-β-hydroxybutyrate, and, thus, ingestion of the novel ketone ester yields only ketone bodies, elevating circulating levels. Oxidative tissues, including muscle, take up ketones *via* monocarboxylate transporters, which leads to their subsequent oxidation in the mitochondria. *B*) Experimental protocol for treadmill and maze testing. Rats were habituated to the 8-arm radial maze for 28 d and the motorized treadmill for 14 d before baseline testing on 3 consecutive days. After baseline tests, rats were allocated to a diet group, rested for 4 d, and pair fed such that rats received the same calories each day irrespective of group. Rats were then rehabituated to the maze and treadmill over 2 d before 5 consecutive days of performance testing. *C*) Macronutrient composition (% kcal) of the Western, carbohydrate, and ketone diets, all of which contained 1.76 kcal/g. CoA, coenzyme A; FFA, free fatty acid; LDH, lactate dehydrogenase.

Here, we questioned whether the metabolism of d-β-hydroxybutyrate, fed in the diet as (*R*)-3-hydroxybutyl (*R*)-3-hydroxybutyrate, could increase physical performance and cognitive function in the rat *in vivo*, and whether any changes were associated with improved high-energy phosphate metabolism.

## MATERIALS AND METHODS

### Animals, diets, exercise capacity, and cognitive function

All studies were approved by Oxford Animal Ethics Review Committees and the Home Office. (*R)*-3-Hydroxybutyl (*R*)-3-hydroxybutyrate and test diets were prepared at the University of Oxford. Young adult male Wistar rats (*n* = 65; starting weight 70 g; Harlan UK, Alconbury, United Kingdom) were housed at 20°C in a humidity-controlled environment on a 12-h light-dark photoperiod in accordance with UK Home Office and U.S. National Institutes of Health guidelines. All rats were initially fed standard laboratory chow *ad libitum* (Rat and Mouse No. 1 Maintenance; SDS, Witham, United Kingdom), which had an Atwater fuel energy of 3.3 kcal/g, with 7.5% of its energy from oil, 17.5% from protein, and 75% from carbohydrate.

Running performance was assessed by using a motorized treadmill (Columbus Instruments, Columbus, OH, USA), whereas an 8-arm radial maze was used to assess working memory, as described previously (15). Rats continued on the standard laboratory chow diet as they were habituated to the maze test (for 1 mo) and treadmill (for the final 7 d of the habituation period) and for baseline testing.

During the maze test, all 8 arms were baited with a food reward (1 ml of 50% sweetened condensed milk, 50% water), which was not replenished during a test. Rats were placed individually in the center of the maze and their movements were monitored. The test was deemed to be completed when a rat had visited all 8 arms or 5 min had elapsed. Time taken to complete the maze, that is, visit all 8 arms, and the number of initial correct decisions taken before making an error, that is, returning to a previously visited arm, were recorded.

Before the exercise test, rats were habituated to a motorized treadmill (Columbus Instruments), running gently at gradually increasing belt velocities. After this 7-d period, all rats were proficient at running on the treadmill for 5 min at a velocity of 10 m/min on a 5° incline. From their first encounter with the treadmill, rats were exposed to an electric stimulus at the rear of the treadmill that delivered a shock of 1.2 mA of current at 3 Hz. A built-in electric fan was used to cool the rats during exercise and to circulate air in the treadmill chamber. Rats subsequently underwent baseline testing, which comprised 3 exercise tests on the treadmill on consecutive days, during which belt velocity was initially set at 10 m/min and increased by 1 m/min^2^ until the rat fatigued, that is, could no longer keep pace with the belt, and at this point the distance run was recorded. After the treadmill test, rats underwent a maze test. After completion of baseline testing, rats were switched to the experimental diets (see Results) for 4 d before the 5 test days (Fig. 1*B*).

Each test day comprised 3 exercise tests (separated by >2 h) and a maze test after the final exercise test. For each day, mean exercise performance is reported here.

### Pyruvate dehydrogenase flux assessment

Pyruvate dehydrogenase (PDH) flux was determined by using hyperpolarized 1-[^13^C] pyruvate as previously described (21). In brief, 1 ml of hyperpolarized tracer was intravenously injected for 10 s into the anesthetized rat and 60 individual cardiac spectra were acquired during 1 min. Cardiac [^13^C] MR spectra were analyzed by using the AMARES algorithm as implemented in the Java-based magnetic resonance user interface (jMRUI) software package (*http://www.jmrui.eu/*) (22). Relative metabolite production was calculated by dividing the maximum metabolite peak area by the maximum pyruvate peak area.

### Cardiac high-energy phosphate metabolism

Rats (*n* = 10 per diet group) continued to be pair-fed test diets for 7 wk after the final day of physical performance testing, without further treadmill or maze testing during this time. For assessment of cardiac energetics, rats were anesthetized by using a 0.5-ml i.p. injection of pentobarbital sodium (200 mg/ml Euthatal; Merial Animal Health, Ltd., Harlow, United Kingdom). The heart was removed and arrested in ice-cold Krebs-Henseleit (KH) buffer (118 mM NaCl, 5.9 mM KCl, 1.2 mM MgSO_4_.7H_2_O, 1.3 mM CaCl_2_.2H_2_O, 0.5 mM Na_2_EDTA, 8 mM glucose, 25 mM NaHCO_3_, 0.2 mM pyruvate, 1.2 mM lactate, 0.1 U/L insulin) and 0.4 mM palmitate bound to 1.5% albumin w/v. Hearts were cannulated *via* the aorta and perfused in a recirculating retrograde Langendorff mode at a constant pressure of 100 mm Hg. KH buffer was aerated with 95% O_2_/5% CO_2_ at a constant working pH of 7.4 at 37°C.

Once the heart was secure and well perfused, any nonmyocardial tissue was carefully trimmed away. A small incision was made in the pulmonary artery to prevent increase in venous pressure. Intraventricular pressure development that resulted from Thebesian artery drainage was minimized by insertion of a polytetrafluoroethylene drain through the apex of the heart. A polyethylene balloon connected to a pressure transducer was inserted into the left ventricle and inflated to produce an end-diastolic pressure of ∼4 mm Hg. Left ventricular developed pressure and heart rate were recorded *via* the pressure transducer connected to a PowerLab/4SP data acquisition system (AdInstruments, Oxford, United Kingdom). Rate pressure product (RPP) was calculated as the product of heart rate and left ventricular developed pressure.

The perfused heart was placed in a 20-mm NMR sample tube and positioned inside the bore of a 11.7-T superconducting magnet. Temperature of the heart and perfusate was maintained at 37°C by using water-jacketed buffer reservoirs and perfusion lines. Intracellular phosphorus metabolites were determined by acquiring partially saturated spectra at a resonant frequency of 202.47 MHz using a 60° pulse and receiver delay of 1 s. Each spectrum consisted of 60 transients, which gave a total acquisition time of 1 min. Spectra were corrected for saturation by comparison with unsaturated spectra, acquired at the end of each experiment by using a 90° pulse and a receiver delay of 2.35 s, with a total acquisition time of 10 min. Signal-to-noise ratio was increased by exponentially multiplying the resultant [^31^P] NMR free induction decay by a line broadening factor of 20 Hz before Fourier transformation.

Individual peak areas were related to absolute intracellular concentrations by assuming cytosolic ATP concentration was equal to 10.6 mM (23). β-ATP peak was used for all ATP, PCr, and inorganic phosphate measurements, as described previously (24, 25).

After initial stabilization of the heart on KH buffer, hearts were perfused with a physiologic buffer for 20 min. To accomplish this, plasma metabolite concentrations were obtained from all rats during the sedentary feeding phase 2 wk before perfusion. Thus, each group received physiologic buffers that contained concentrations of palmitate (prebound to 1.5% w/v albumin), β-hydroxybutyrate, acetoacetate, and lactate tailored to mimic the circulating *in vivo* metabolite concentrations, in addition to the standard KH buffer. Thus, concentrations were as follows: Western diet: 0.41 mM palmitate, 0.3 mM β-hydroxybutyrate, 0.1 mM acetoacetate, and 1.2 mM lactate; carbohydrate diet: 0.29 mM palmitate, 0.3 mM β-hydroxybutyrate, 0.1 mM acetoacetate, and 1.2 mM lactate; and ketone diet: 0.36 mM palmitate, 0.5 mM β-hydroxybutyrate, 0.1 mM acetoacetate, and 2 mM lactate.

During perfusion with physiologic buffer, workload was increased by the coronary infusion of 10 nM isoproterenol, a β-adrenergic receptor agonist. The optimal isoproterenol concentration to increase workload without damaging the heart was calculated in previous experiments (24). Damage was defined as the decrease in contractile function (*i.e.,* RPP). The 10 nM isoproterenol solution was administered for 5 min *via* an external line fed into the main buffer flow above the ascending aorta. Cardiac function was monitored and short 1-min [^31^P] spectra were recorded throughout.

### Cardiac and skeletal muscle protein levels

Levels of UCP3 were measured in heart and skeletal muscle samples by immunoblotting, as described previously (26, 27), by using a polyclonal rabbit anti-UCP3 antibody (Millipore, Billerica, MA, USA) at a concentration of 1:1000 in 5% milk Tris-buffered saline (TBS)-Tween. Glucose transporter 4 (GLUT4) levels were measured as described previously (23) by using a rabbit anti-GLUT4 antibody kindly provided by Prof. G. D. Holman (University of Bath, Beth, United Kingdom) at a concentration of 1:4000 in 5% milk TBS-Tween. Citrate synthase levels were measured by using a rabbit anti-CS antibody (Millipore) at a concentration of 1:1000 in 5% milk TBS-Tween. Peroxisome proliferator-activated receptor-γ coactivator 1α (PGC1α) levels were measured by using a rabbit anti-PGC1α antibody (Santa Cruz Biotechnology, Santa Cruz, CA, USA) at a concentration of 1:1000 in 5% milk TBS-Tween. Monocarboxylate transporter 1 (MCT1) levels were measured by using a rabbit anti-MCT1 antibody (Santa Cruz Biotechnology) at a concentration of 1:2500 in 5% milk TBS-Tween. The secondary antibody in all cases was a goat anti-rabbit IgG antibody conjugated to horseradish peroxidase, which was used at concentrations between 1:3500 and 1:2000 in 5% milk TBS-Tween. Membranes were incubated with ECL plus detection solution (Amersham Biosciences, Little Chalfont, United Kingdom) and were exposed to X-ray film to visualize protein bands, which were quantified by using Un-Scan-It Gel 6.1 (Silk Scientific, Orem, UT, USA).

### Plasma metabolite concentrations

Plasma concentrations of acetone, *R*-1,3-butanediol, and (*R)*-3-hydroxybutyl (*R*)-3-hydroxybutyrate were determined by using GC-MS analyses. Acetone concentrations were determined by using headspace analysis of the volatile compounds from a 20-µl amount of neat plasma samples (28). H_6_-Acetone was used as an internal standard to quantify plasma acetone concentrations. (*R)*-3-Hydroxybutyl (*R*)-3-hydroxybutyrate and 1,3-butanediol were analyzed as their trimethylsilyl ethers in electron impact mode by GC-MS. Neutralized perchloric acid plasma extracts (20 μl) were brought to dryness under a stream of N_2_ and were reacted with 100 μl Tri-Sil TBT reagent (Pierce, Rockford, IL, USA) to form the trimethylsilyl ethers (29). Samples were sealed and heated to 60°C for 5 min. One microliter of derivatized sample extracts was analyzed by capillary column GC-MS. 1,3-Butanediol was quantified by using 1,4-butanediol as an internal standard added to the samples before sample extraction. (*R)*-3-Hydroxybutyl (*R*)-3-hydroxybutyrate was quantified by using an external standard procedure by spiking plasma with the analyte over a concentration range from 1 to 100 μM.

### Cardiac and hepatic metabolomic analysis

Heart and liver tissue from rats that were fed the 3 diets were analyzed by a combination of [^1^H] NMR spectroscopy and GC-MS as part of a metabolomic strategy to assess metabolic differences in the 3 tissues, as described previously (30).

### Statistics

ANOVA with repeated measures was used to determine whether there were differences between the diet groups, and Bonferroni *post hoc* independent unpaired Student’s *t* tests were used to determine differences between diet groups. Paired Student’s *t* tests were used to determine within-group differences between baseline and test week.

## RESULTS

After a habituation period and baseline treadmill and maze testing (Fig. 1*B*), rats were fed one of 3 isocaloric (1.76 kcal/g) diets: *1*) a fat-rich Western diet in which 30% of kcal came from added palm oil (*n* = 20); *2*) a high-carbohydrate diet in which 30% of kcal came from added corn starch (*n* = 10); or *3*) a ketone diet in which 30% of kcal came from (*R)*-3-hydroxybutyl (*R*)-3-hydroxybutyrate (*n* = 20; Fig. 1*C*). Rats were pair fed, such that rats fed the Western and carbohydrate diets received the same number of calories as those consumed by the ketone diet–fed rats on the previous day. Before introduction of the test diet, all rats consumed 33 ± 2 kcal/d from normal rodent chow. Over a 5-d performance period, total daily calorie intake was 39 ± 2 kcal/rat/d. Thus, ketone diet–fed rats consumed 2.5 g of (*R)*-3-hydroxybutyl (*R*)-3-hydroxybutyrate per d, which was equivalent to 11.7 g/kg body weight/d.

After 9 d, plasma levels of d-β-hydroxybutyrate in ketone diet–fed rats were double (*P* < 0.001) those of rats fed the Western diet, whereas no (*R)*-3-hydroxybutyl (*R*)-3-hydroxybutyrate could be detected in any plasma sample. (*R*)-1,3-Butanediol concentrations, at 70–110 μM, were 15% of d-β-hydroxybutyrate concentrations, and acetone levels were 60–80 μM, which demonstrated that the (*R)*-3-hydroxybutyl (*R*)-3-hydroxybutyrate had been hydrolyzed completely and butanediol had been converted to ketone bodies. Plasma cholesterol and triglyceride levels in the ketone diet–fed rats were 37% (*P* < 0.001) and 59% (*P* < 0.01) lower, respectively, than in rats fed the Western diet, which was consistent with the inability of the liver to oxidize ketone bodies as a result of the absence of 3-oxoacid-CoA transferase (EC 2.8.3.5). Plasma glucose, free fatty acids, and lactate dehydrogenase levels were the same for all rats.

**Figure 2.**
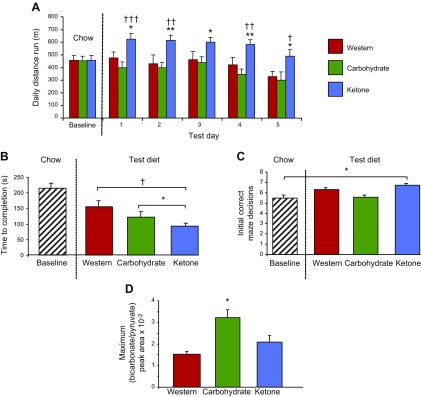
Effect of feeding the ketone diet for 9 d on physical performance, cognitive function, and cardiac PDH flux. *A*) Effect of diet on distance run by rats on a motorized treadmill on 5 consecutive days. *B*) Rats that were fed the ketone diet were faster at completing the 8-arm radial maze than were rats that were fed the Western or carbohydrate diets. *C*) Rats that were fed the ketone diet made more correct decisions than at baseline. *D*) Male Wistar rats (*n* = 5/diet) were fed one of the 3 diets for 9 d and *in vivo* PDH flux—determined by using hyperpolarized [^13^C] MR—was found to be higher only in the carbohydrate-fed rat hearts. *P < 0.05, **P < 0.01 *vs*. rats fed the Western diet; ^†^*P* < 0.05, ^††^*P* < 0.01, ^†††^*P* < 0.001 *vs*. rats fed the carbohydrate diet.

At baseline, when consuming chow, rats with matching running capacities were allocated to the 3 diet groups, with an average running distance of 456 ± 38 m/d. On each of the final 5 d on the diets, ketone diet–fed rats ran 32% (*P* < 0.05) further than did the Western diet– and carbohydrate diet–fed rats (**Fig. 2*A***). Ketone diet–fed rats were the only group that increased running performance from baseline, averaging 22% (*P* < 0.05) further each day over the 5 d.

After the third treadmill run each day, rats performed an 8-arm radial maze test (31). Although the time to complete the test was the same for all rats at baseline, ketone diet–fed rats were 38% (*P* < 0.05) faster at completing the radial maze than Western diet– and carbohydrate diet–fed rats (Fig. 2*B*). Cognitive function, measured by the number of correct arm choices made, was the same for the 3 diet groups at baseline. Ketone diet–fed rats made more correct decisions before making a mistake during the test week than at baseline, whereas rats that were fed the other diets did not show a significant difference in performance (Fig. 2*C*).

End body weights were similar for the 3 diet groups at 218 ± 4 g, 213 ± 8 g, and 209 ± 6 g for the Western diet–, carbohydrate diet–, and ketone diet–fed rats, respectively. Epididymal fat pad weights [an index of visceral adiposity (32)] were 40% (*P* < 0.05) lower in the ketone diet–fed rats compared with those fed the Western diet. *In vivo* hyperpolarized [^13^C] MR spectroscopy (21) revealed that rats that were fed the carbohydrate diet had 2-fold greater cardiac PDH flux than did rats that were fed either the Western or ketone diets (Fig. 2*D*). Thus, the ketone diet may provide energy in conditions of insulin resistance when PDH is inhibited, such as diabetes, but also trauma, infection, heart failure, and Alzheimer’s disease (21, 33, 34).

Three groups of 10 rats continued to be pair fed the diets for a total of 66 d, during which time they increased normally in weight and by a similar amount (∼35%). End body weights were similar, at 297 ± 10 g, 292 ± 9 g, and 277 ± 17 g for Western diet–, carbohydrate diet–, and ketone diet–fed rats, respectively. Food intake during the 66 d of feeding was 53 ± 1 kcal/rat/d. Thus, ketone diet–fed rats consumed 3.4 g of (*R)*-3-hydroxybutyl (*R*)-3-hydroxybutyrate daily, equivalent to 13.7 g/kg body weight/d.

**Figure 3.**
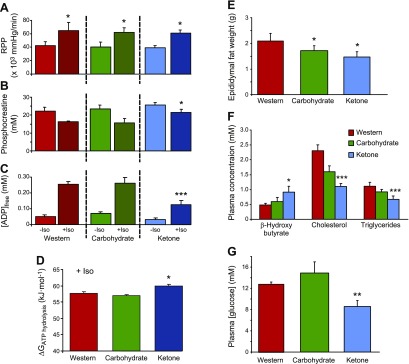
Effect of feeding the ketone diet for 66 d on myocardial contractile function and high-energy phosphate levels, epididymal fat pad weights, and plasma metabolite concentrations. *A*) RPP, a measure of workload, was increased by 34% using an infusion of 10 nM isoproterenol (Iso) for 5 min in each rat heart. *P < 0.05 *vs*. –Iso. *B*) PCr remained higher in ketone-fed rat hearts during the increased workload compared with hearts from rats that were fed either of the other 2 diets. *P < 0.05 *vs*. rats fed the Western and carbohydrate diets. *C*) Free ADP concentration was lower in ketone-fed rat hearts during the increased workload compared with hearts from rats that were fed either of the other 2 diets. ***P < 0.001 *vs*. rats fed the Western and carbohydrate diets. *D*) Free energy of ATP hydrolysis was higher in hearts from ketone-fed rats during the high workload. *P < 0.05 *vs*. rats fed the Western and carbohydrate diets. *E*) Epididymal fat pad weights were lower in rats that were fed the ketone and carbohydrate diets. *P < 0.05. *F*) Ketone diet increased plasma concentrations of d-β-hydroxybutyrate and lowered plasma cholesterol and triglycerides. *P < 0.05, ***P < 0.001 *vs*. rats fed the Western diet. *G*) Ketone diet lowered plasma glucose concentrations. **P < 0.01, *vs*. rats fed the Western diet.

To determine whether the ketone diet increased the free energy available from ATP hydrolysis (*∆G_ATP hydrolysis_*), hearts were isolated and perfused for [^31^P] MR spectroscopy experiments (23). Heart rates and developed pressures were continuously monitored to allow the calculation of RPPs. During perfusion, RPPs increased by 34% (*P* < 0.05) in all hearts for 5 min after addition of 10 nM isoproterenol, a β-adrenergic receptor agonist (**Fig. 3*A***). Before isoproterenol infusion, [PCr] and calculated free adenosine diphosphate concentrations ([ADP]_free_) were the same for all rat hearts (Fig. 3*B*, *C*). During isoproterenol perfusion, [PCr] was 32% higher (*P* < 0.05) and [ADP]_free_ was 50% lower (*P* < 0.001) in ketone diet–fed rat hearts compared with Western diet– and carbohydrate diet–fed rat hearts. ATP concentrations and pH remained the same for all rat hearts, but the *∆G_ATP hydrolysis_* was 2.6 kJ⋅mol^−1^ (*P* < 0.05) greater during the infusion of isoproterenol in hearts from rats that were fed the ketone diet compared with those from rats that were fed Western and carbohydrate diets (Fig. 3*D*).

Heart weights were the same for all the rats; however, epididymal fat weights were 18% (*P* < 0.05) lower in the carbohydrate diet–fed rats and 30% (*P* < 0.05) lower in the ketone diet–fed rats than in the Western diet–fed rats after 66 d on diet (Fig. 3*E*). Plasma levels of β-hydroxybutyrate in ketone diet–fed rats remained double those of rats on the Western diet (Fig. 3*F*). No (*R)*-3-hydroxybutyl (*R*)-3-hydroxybutyrate could be detected in any plasma sample. Plasma concentrations of cholesterol were 52% lower—with significant decreases in both HDL and LDL—triglycerides were 40% lower, and glucose was 33% lower (Fig. 3*G*) in rats that were fed the ketone diet than in rats that were fed the Western diet. Citrate synthase activity was the same in all rat heart tissue, which suggested that there was no change in mitochondrial density as a consequence of diet. Heart and skeletal muscle protein levels of UCP3, GLUT4, citrate synthase, PGC1α, and MCT1 were unaltered after 66 d on any diet.

**Figure 4.**
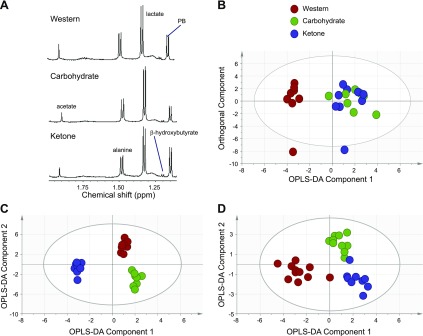
Metabolite analysis of liver and heart after 66 d feeding the ketone diet. *A*) Expanded region of the [^1^H] NMR spectra from liver tissue from animals that were fed the 3 diets to demonstrate the increase in β-hydroxybutyrate. *B*) Pattern recognition model [orthogonal partial least squares discriminate analysis (OPLS-DA)] of the total fatty acid complement of liver tissue as measured by GC free induction decay, demonstrating the differences between animals that were fed either Western, carbohydrate, or ketone diets (*R*^2^, 40%; Q^2^, 33%). *C*) Pattern recognition model of the aqueous metabolites detected by high-resolution [^1^H] NMR spectroscopy of liver tissue extracts from rats that were fed the 3 diets using OPLS-DA (*R*^2^, 83%; Q^2^, 59%). *D*) Pattern recognition model (OPLS-DA) distinguishing the total fatty acid complement of heart tissue from rats that were fed the 3 diets (*R*^2^, 64%; Q^2^, 68%). PB, phenobarbital.

Metabolic differences in liver and heart tissue from rats that were fed the 3 diets for 66 d were determined by using [^1^H] NMR spectroscopy and GC-MS (30). In the liver, proton NMR spectroscopy detected a 2-fold higher level of β-hydroxybutyrate from rats that were fed the ketone diet compared with the other 2 diets (*P* < 0.01; **Fig. 4*A*** and **Table 1**). Orthogonal partial least squares discriminate analysis of the liver fatty acid profile allowed the 3 groups to be distinguished by using pairwise comparisons (Fig. 4*B*).

**TABLE 1.** Summary of fold changes for key metabolite differences discriminating the 3 diet groups

Metabolite	Ketone *vs*. carbohydrate	Western *vs.* carbohydrate	Ketone *vs*. Western
Liver			
β-Hydroxybutryate	2.00*	1.00	1.36**
ATP	1.65*	1.00	1.66***
Adenosine	0.76	0.96	0.79
Lactate	1.30	1.96**	0.66
Alanine	0.55	1.76	0.31
C16:0	0.95	1.07	0.88**
C16:1 (*cis*-9)	0.71	0.28***	2.52***
C17:1 (*cis*-10)	0.96	Not detected in Western diet–fed rats***	Not detected in Western diet–fed rats***
C18:1 (*cis*-9)	1.09	1.57*	0.69**
C18:2 (*cis*-9, 12)	1.02	1.17*	0.87***
C22:6 (*cis*-4, 7, 10, 13, 16, 19)	1.08	0.66***	1.63***
Heart			
ATP	1.03	0.84	1.23
Lactate	0.99	1.88	0.53
Taurine	1.16	1.51	0.76
C16:0	1.18**	1.08	1.09
C16:1	1.15	0.29***	3.93***
C17:1 (*cis*-10)	4.22**	3.58	1.18
C18:1 (*cis*-9)	1.03	0.40***	1.19***
C18:2 (*cis*-9, 12)	0.96	0.81***	1.19***
C22:6 (*cis*-4, 7, 10, 13, 16, 19)	0.87	1.24**	0.70***

Metabolites were first identified by multivariate statistics [orthogonal partial least squares discriminate analysis (OPLS-DA)]. Fold changes are an average of area under the peak for each individual metabolite in the relevant spectrum or chromatogram. Data was then compared by using ANOVA, followed by Tukey’s *post hoc* test. Where no asterisk is present, no difference was found in the ANOVA test after OPLS-DA. *P < 0.01, **P < 0.05, ***P < 0.005.

Comparison of all 3 groups demonstrated that the Western diet group was most different from the other 2 groups, having 7% higher saturated fat (*P* < 0.001). Compared with the Western diet, rats that were fed the ketone diet had decreased amounts of liver palmitate, oleate, C18:2 and increased concentrations of C22:6, C17:1, and C16:1 fatty acids (Table 1). [^1^H] NMR spectroscopy of the aqueous liver metabolites distinguished between all 3 groups (Fig. 4*C*). Ketone diet–fed rats had the most distinct metabolic changes, with increased concentrations of ATP, lactate, and β-hydroxybutyrate and decreased glucose, alanine, and adenosine (Table 1).

In the heart, only small differences were detected in the aqueous fraction of the tissue from ketone diet–fed animals, with increased ATP and decreased lactate and taurine. Total fatty acid content distinguished heart tissue according to the 3 diets (Fig. 4*D*), including a relative decrease in monounsaturated and polyunsaturated fatty acids in the heart from animals that were fed the Western diet compared with the other 2 diets (Table 1).

## DISCUSSION

The possible benefits of mild ketosis for the improvement of performance and cognition during endurance exercise as a result of the thermodynamic advantage afforded by ketone body oxidation over the oxidation of alternative substrates have been hypothesized (3, 6). Here, we found that a diet that contained a novel ketone ester [30% kcal from (*R*)-3-hydroxybutyl (*R*)-3-hydroxybutyrate] elevated circulating β-hydroxybutyrate concentrations in rats and lowered plasma cholesterol, triglyceride, and glucose levels compared with pair-fed, isocaloric diets in which the ketone ester was replaced by fat or carbohydrate. Supplementation with a ketone ester improved running performance on a motorized treadmill and working memory during a maze test. Mechanistically, ketone ester supplementation was found to increase the free energy available from ATP hydrolysis in the isolated, perfused rat heart under conditions of increased work.

In humans, ketosis can occur in the pathologic setting of type 1 diabetes, where insulin deficiency results in a release of free fatty acids from adipose tissue that exceeds the capacity of other tissues to metabolize them, driving ketone body synthesis by hepatic mitochondria (3). In this situation, elevation of blood ketone levels to ≥20 mM (35) depletes blood bicarbonate and acidifies the blood to pH 6.9 (3). Physiologic ketosis, which contrasts with diabetic ketoacidosis, is a normal physiologic state with no known adverse medical consequences (3). In humans, ketosis will result from prolonged starvation and is accompanied by high circulating free fatty acid levels (7, 36). Alternatively, ketosis can be achieved *via* the so-called ketogenic diet, in which 80% of daily calorie intake is typically derived from fat, with 15% from protein and 5% from carbohydrate (37). With adjustment to the ketogenic diet, whole-body glucose oxidation falls and plasma glucose levels can be maintained, albeit at lower concentrations than in carbohydrate diet–fed individuals (12). Usefulness of the ketogenic diet in achieving long-term ketosis, however, is limited as a result of the elevation of blood cholesterol and LDL cholesterol, which predisposes the individual to atherosclerosis (11). Moreover, the elevation of plasma free fatty acid levels that results from lipolysis of adipose tissue may offset many of the benefits of ketone metabolism *via* activation of peroxisome proliferator-activated receptors and the possible uncoupling of oxidative phosphorylation at the mitochondria (13, 14, 38).

(*R*)-3-Hydroxybutyl (*R*)-3-hydroxybutyrate has been shown to be safe in rats with 28 d of feeding (19), and has similarly been found to be well-tolerated in humans (20). Here, we studied exercise and cognitive performance in rats with relatively short-term feeding of the ketone ester (9 d), but continued to feed a subgroup of rats for a total of 66 d. Thus, we have extended the time during which (*R*)-3-hydroxybutyl (*R*)-3-hydroxybutyrate has been fed at 30% caloric intake with no adverse effects. It has been shown that, after 14 d, supplementation with the ester lowered blood glucose and insulin levels, which indicates an improvement in insulin sensitivity (3, 39). Because insulin increases sterol regulatory binding protein (SREBP) in the liver, which increases expression of genes associated with cholesterol synthesis, it was proposed that ketone ester supplementation would also have antilipidemic effects (3). We found short-term effects of ketosis on plasma cholesterol and triglyceride levels, effects that were sustained after longer-term supplementation. This is in contrast with hyperlipidemia produced by the ketogenic diet (11), which indicates that ketone ester supplementation is a viable strategy to support long-term ketosis and avoid the risk of atherosclerosis. Moreover, the lipidomic profile of liver and cardiac tissue after long-term feeding distinguished the ketone diet–fed rats from those fed the other diets tested in this study, while higher β-hydroxybutyrate levels were associated with higher ATP levels in the livers of ketone diet–fed rats.

(*R*)-3-Hydroxybutyl (*R*)-3-hydroxybutyrate has been shown to have antilipidemic effects in rats and humans (40) and to increase mitochondrial biogenesis and UCP1 expression in brown adipose tissue of mice (41), as well as to decrease appetite in rats and mice (39, 41). As a precaution against possible palatability issues or appetite-suppression effects associated with ketone ester feeding, rats that were allocated to the carbohydrate and Western diets in this study were pair fed, receiving the same calories each day as their ketone-supplemented counterparts received the day before. Of note, however, calorie consumption remained high in the ketone-supplemented rats throughout the study. Caloric intake increased, as expected, during the exercise-testing week in response to increased energy demand, but ketone intake (corrected for body mass) increased further during the prolonged sedentary period after exercise testing. Use of a pair-feeding regime and isocaloric diets was a major strength of this study.

In agreement with our hypothesis, supplementation with (*R*)-3-hydroxybutyl (*R*)-3-hydroxybutyrate for 5 d improved running performance in rats during an endurance test on a motorized treadmill. Performance was elevated both above baseline running performance and above that of rats that were pair fed isocaloric diets with ketone ester replaced with fat or carbohydrate. In previous studies, using this protocol, we have found that provision of a high-fat diet (55% of kcal from fat) worsened running performance in rats (15), as did a diet in which long-chain triglycerides (46% kcal) were provided, although rats that consumed medium-chain triglycerides (46% kcal; associated with increased ketone body production) were not affected to the same extent (42). Researchers have proposed the use of the ketogenic diet as a training aid because of its effects in elevating muscle fatty acid oxidation rates (43). Others, meanwhile, have suggested that the well-documented weight loss engendered by the ketogenic diet might hold some benefits for athletes in decreasing fat mass while conserving muscle and, thus, strength (37). Although increased fat availability does result in elevated whole-body and muscle fat use during submaximal exercise, there is no consistent evidence that the ketogenic diet improves endurance performance (44–46). In fact, in the short term, at least, the ketogenic diet has been seen to impair whole-body exercise efficiency (16) and cardiac high-energy phosphate metabolism (17).

Mechanistically, ketone ester supplementation would be expected to improve performance because of the thermodynamic advantages of ketone oxidation (3, 6), though it may also support increased muscle work during physiologic stress by sparing alternative energy sources, particularly glycogen, as muscle glycogen content correlates with endurance exercise in humans (47). In rats that were supplemented with (*R*)-3-hydroxybutyl (*R*)-3-hydroxybutyrate for 66 d, metabolic profiles of liver and heart tissue were altered compared with those of rats that were fed the other diets. Distinct features of ketone diet–fed rats included elevated ATP levels in liver and, in the heart, lower lactate levels, which may suggest a decreased reliance on glycolysis and, thus, myocardial glycogen reserves, albeit under sedentary conditions. If this were indeed the case, exogenous ketone supplementation would represent a novel metabolic state of ketosis with replete muscle glycogen reserves.

The increased physical performance observed in this study while feeding a ketone diet is consistent with the increase in hydraulic efficiency seen in the working rat heart perfused with ketone bodies *vs*. glucose alone (5), and was reflected in our perfusion studies after long-term ketone supplementation. A strength of the study design was the use of physiologically relevant perfusion buffers during the measurement of cardiac energetics in isolated hearts before and during conditions of increased work after isoproterenol supplementation. Use of these buffers allowed us to reproduce *ex vivo* the substrate balance available to the hearts of the animals that are under physiologic stress *in vivo,* which thereby demonstrates an enhanced Δ*G_ATP hydrolysis_* during increased work. Increase in physical performance in muscle, therefore, is a result of the increased ATP produced when C_2_ units from d-β-hydroxybutyrate were oxidized in the citric acid cycle, as opposed to C_2_ units derived from pyruvate or from fat, as well as the lower intracellular [ADP]. During intense exercise, a switch in fuel use occurs, with the oxidation of glycogen-derived pyruvate increasing at higher intensities because of limitations on the rate of muscle and mitochondrial uptake of fatty acids and the increased oxygen demand of fatty acid oxidation compared with pyruvate (48). In the hearts of ketone diet–fed rats, however, PDH flux was lower compared with the carbohydrate diet–fed group, which suggests that exogenous ketones alter the normal hierarchy of substrate use during exercise, with ketones oxidized in preference to pyruvate when available. This would make sound evolutionary sense, as ketones cannot be stored and would ordinarily be present under conditions of limited substrate availability, that is, starvation.

The preferential oxidation of ketones over both fatty acids and pyruvate suggests that exogenous ketone supplementation may be beneficial during conditions in which normal substrate use is compromised. Recent reports have suggested that increased ketone use underpins a key metabolic adaptation in advanced heart failure in both rodents (49) and humans (50). Whereas the healthy human heart relies primarily on fatty acid oxidation to provide the significant daily requirements of ATP to support normal pump function, in the hypertrophied and failing heart, a remodeling of mitochondrial energy metabolism occurs that results in a decreased myocardial capacity for fatty acid oxidation (51), the so-called fetal shift (52). In human heart failure, however, circulating plasma free fatty acids are elevated (53), secondary to increased sympathetic activation and—alongside a decreased capacity for fatty acid oxidation—can result in intramyocardial lipid accumulation (54) and myocardial insulin resistance (23, 55). The failing heart is characterized by poor myocardial energetics, which predict mortality (56), underlaid by an impaired capacity to either oxidize fatty acids or to take up glucose in response to insulin stimulation (57). The recent finding of a switch toward increased ketone use in end-stage human heart failure (50) is intriguing in that ketones seem to offer a means of circumventing impaired substrate use, with an increase in the myocardial expression of β-hydroxybutyrate dehydrogenase 1 in mice with heart failure (49). In human heart failure, mild ketosis may occur as a result of increased hepatic ketogenesis driven by elevation in free fatty acids that occurs secondary to the hyperadrenergic state. Ketone ester supplementation may therefore improve energetics in the failing heart, increasing availability of the substrate of choice, and may hold further benefits for the heart and other tissues in conditions of insulin resistance by providing an alternative fuel or improving insulin sensitivity by decreasing plasma free fatty acids (3).

The ability of ketosis to improve cognitive performance during a maze test of working memory suggests that the brain readily uses ketone bodies in preference to glucose (8, 58, 59). A ketogenic diet increased human brain PCr, as observed using [^31^P] MR spectroscopy (60), and, hence, increased the *ΔG_ATP hydrolysis_* by virtue of the near equilibrium of the creatine kinase reaction (61). However, hyperlipidemia (11) and the threat of vascular disease produced by feeding high-fat, low-carbohydrate diets has limited the use of ketogenic diets to those age <17 yr. A ketone ester diet would overcome these difficulties. In a mouse model of Alzheimer’s disease, the ketone ester lessened amyloid β-peptide and hyperphosphorylated τ deposition, which lessened anxiety and improved cognition (62), whereas similar improvements in cognition were observed in a patient with Alzheimer’s disease who was treated with (*R*)-3-hydroxybutyl (*R*)-3-hydroxybutyrate over 20 mo (63). Ketone supplementation may also have therapeutic benefits in cases of traumatic brain injury, during which opening of the mitochondrial permeability transition pore occurs as a result of a decrease in brain phosphorylation energy and further impairs ATP synthesis (64). The ketogenic diet has been shown to be neuroprotective in rodent models of traumatic head injury, but has limited use in humans because of the time (days) needed to elevate blood ketone levels (65), whereas a rapid elevation in blood ketone levels could be achieved *via* (*R*)-3-hydroxybutyl (*R*)-3-hydroxybutyrate.

## CONCLUSIONS

Mild ketosis is useful in the treatment of drug-resistant epilepsy. Studies in cells (33), animals (66), and patients (10, 59) suggest that it may be useful in common neurodegenerative diseases, such as Parkinson’s and Alzheimer’s diseases, as well as in insulin-resistant states, such as trauma, heart failure, and diabetes (67). Our results show that mild ketosis markedly improved both physical and cognitive performance in the rat, suggesting that feeding an energy-efficient ketone diet could be used to treat human metabolic disorders.

## Abbreviations

*∆G_ATP hydrolysis_*: free energy available from ATP hydrolysis
GLUT4: glucose transporter 4
KH: Krebs-Henseleit
MCT1: monocarboxylate transporter 1
PCr: phosphocreatine
PDH: pyruvate dehydrogenase
PGC1α: peroxisome proliferator-activated receptor-γ coactivator 1α
RPP: rate pressure product
TBS: Tris-buffered saline
UCP: uncoupling protein
